# Enzyme therapy and immune response in relation to CRIM status: the Dutch experience in classic infantile Pompe disease

**DOI:** 10.1007/s10545-014-9707-6

**Published:** 2014-04-09

**Authors:** Carin M. van Gelder, Marianne Hoogeveen-Westerveld, Marian A. Kroos, Iris Plug, Ans T. van der Ploeg, Arnold J. J. Reuser

**Affiliations:** 1 Department of Pediatrics, Division of Metabolic Diseases and Genetics, Center for Lysosomal and Metabolic Diseases, Erasmus MC University Medical Center, Rotterdam, The Netherlands; 2 Department of Clinical Genetics, Center for Lysosomal and Metabolic Diseases, Erasmus MC University Medical Center, Dr Molewaterplein 50, 3015 GE Rotterdam, The Netherlands

## Abstract

**Background:**

Enzyme-replacement therapy (ERT) in Pompe disease—an inherited metabolic disorder caused by acid α-glucosidase deficiency and characterized in infants by generalized muscle weakness and cardiomyopathy—can be complicated by immune responses. Infants that do not produce any endogenous acid α-glucosidase, so-called CRIM-negative patients, reportedly develop a strong response. We report the clinical outcome of our Dutch infants in relation to their CRIM status and immune response.

**Methods:**

Eleven patients were genotyped and their CRIM status was determined. Antibody formation and clinical outcome were assessed for a minimum of 4 years.

**Results:**

ERT was commenced between 0.1 and 8.3 months of age, and patients were treated from 0.3 to 13.7 years. All patients developed antibodies. Those with a high antibody titer (above 1:31,250) had a poor response. The antibody titers varied substantially between patients and did not strictly correlate with the patients’ CRIM status. Patients who started ERT beyond 2 months of age tended to develop higher titers than those who started earlier. All three CRIM-negative patients in our study succumbed by the age of 4 years seemingly unrelated to the height of their antibody titer.

**Conclusion:**

Antibody formation is a common response to ERT in classic infantile Pompe disease and counteracts the effect of treatment. The counteracting effect seems determined by the antibody:enzyme molecular stoichiometry. The immune response may be minimized by early start of ERT and by immune modulation, as proposed by colleagues. The CRIM-negative status itself seems associated with poor outcome.

**Electronic supplementary material:**

The online version of this article (doi:10.1007/s10545-014-9707-6) contains supplementary material, which is available to authorized users.

## Introduction

Immune responses are common in lysosomal storage disorders (LSDs) in which enzyme-replacement therapy (ERT) is applied (Brooks et al 2003; Wang et al 2008). The aim of ERT is to correct the enzyme deficiencies in LSDs by intravenous infusion of recombinant human enzymes. ERT is now available for Gaucher disease, Fabry disease, the mucopolysaccharidoses (MPS I, II and VI), and for Pompe disease. Immune responses have been seen in all these diseases (Brooks et al 2003; Wang et al 2008).

Pompe disease (glycogen storage disease type II, acid maltase deficiency, OMIM # 232300) is a lysosomal storage disorder caused by mutations in the acid α-glucosidase gene (*GAA*, EC 3.2.1.20) (Hirschhorn and Reuser 2001; van der Ploeg and Reuser 2008). The disease is autosomal recessive and has a broad clinical spectrum (Güngör and Reuser 2013). At the severe end of the spectrum, patients with classic infantile Pompe disease present with muscle weakness, hypertrophic cardiomyopathy and respiratory insufficiency in the first few months of life. If untreated, they usually succumb to cardio-respiratory insufficiency before the end of their first year (van den Hout et al 2003; Kishnani et al 2006a). Their rapid demise is caused by a virtually total deficiency of acid α-glucosidase activity. Treatment with alglucosidase alfa reverses the cardiomyopathy, improves motor function, and extends the survival of patients with classic infantile Pompe disease (Van den Hout et al 2000, van den Hout et al 2004; Klinge et al 2005a; Klinge et al 2005b; Kishnani et al 2006b,  2007; Nicolino et al 2009; Kishnani et al 2009; Chakrapani et al 2010).

Over 95 % of affected infants receiving ERT develop antibodies to alglucosidase alfa (Kishnani et al 2010). In classic infantile Pompe disease, a distinction is often made between CRIM-negative patients (cross reactive immunological material) who lack any form of endogenous acid α-glucosidase and CRIM-positive patients who synthesize some catalytically inactive acid α-glucosidase. High and sustained antibody titers were reported to occur mainly in CRIM-negative patients and to be associated with a poor clinical outcome (Kishnani et al 2010; Banugaria et al 2011). Here we report on the immune response to ERT in 11 patients with classic infantile Pompe disease who were treated for up to 13 years. We examined 1.) the relationship between antibody formation and the patients’ CRIM status and 2.) the impact of antibody formation on the patients’ clinical outcome.

## Materials and methods

### Patients

We describe 11 patients with classic infantile Pompe disease who received ERT in our hospital between 1999 and 2012. Classic infantile Pompe disease was defined as symptoms of muscle weakness within 6 months after birth; hypertrophic cardiomyopathy (left ventricular mass index (LVMI) > +2SD (>75 g/m^2^) (Poutanen and Jokinen 2007)); less than 1 % acid α-glucosidase activity in fibroblasts; and severe mutations in both *GAA* alleles. Activity assays and mutation analysis were performed as described previously (Kroos et al 1997, 2007). All patients participated in consecutive trials investigating the safety and efficacy of ERT (20 mg/kg every other week to 40 mg/kg weekly). Initially, four patients received recombinant human α-glucosidase from the milk of transgenic rabbits (Van den Hout et al 2000). From 2004 onward, all patients were treated with alglucosidase alfa. The Institutional Review Board approved all studies and the parents of all patients gave written informed consent.

### CRIM status

Two methods were used to determine the patients’ CRIM status. Cultured skin fibroblasts from the patients were used in the first method. They were grown in Dulbecco’s modified Eagle’s medium (DMEM) supplemented with 10 % fetal calf serum (FCS) and antibiotics, and were harvested with trypsin 1 week after reaching confluence. Frozen cell pellets were lysed in 10 mM phosphate-buffered saline (PBS) pH 7.0 containing 1 % Triton-X100 and protease inhibitors. A 10 μL aliquot containing 100 μg protein was mixed with 10 μL sample buffer (NuPAGE, LDS sample buffer, Life Technology, with 4 % SDS instead of 0.84 %) and applied to SDS-PAGE (Bio-Rad Criterion XT 4–12 % MOPS gel system). After blotting onto nitrocellulose the various molecular forms of acid α-glucosidase were visualized with polyclonal rabbit antibodies against recombinant-human acid α-glucosidase and goat anti-rabbit IRDye 680LT as secondary antibody using an Odyssey infrared imager (LI-COR Biosciences).

The second method for determining the CRIM status was based on transient expression of *GAA* cDNA constructs containing the patients’ pathogenic mutations in HEK293T cells (one mutation per construct). The activity of acid α-glucosidase in these cells and in the culture medium was measured with the artificial substrate 4-methylumbelliferyl-α-D-glucopyranoside (MUGlc, Sigma) at 72 h after transfection. The biosynthetic forms of acid α-glucosidase were separated by SDS-PAGE and visualized by immunoblotting (Hermans et al 1998).

### Antibody detection

Blood samples for antibody titer measurements were drawn before the start of ERT and every 3 months thereafter just before the start of enzyme infusion. The sera were stored at -20°C. An enzyme-linked immunosorbent assay (ELISA) was used to determine the titers. To this end a 96-well plate (Nunc, F96 Maxisorp, Denmark) was coated with 50 μL/well alglucosidase alfa in a concentration of 5 μg/mL, diluted in PBS (pH 7.4), and incubated for 2 h at room temperature under continuous shaking. Plates were blocked overnight at 4°C with a solution of bovine serum albumin (BSA; Sigma A7030) in PBS (1 % weight/volume). The plates were rinsed six times at room temperature with 200 μL washing buffer (0.05 % Tween-20 in PBS). Plates were then incubated with 50 μL of five-fold serial dilutions of patients’ sera for 1 h during shaking. Samples were diluted from 50 to 3,906,250 fold in dilution buffer (BSA/PBS containing 0.05 % Tween-20). Sera from healthy individuals were used as negative controls, and rabbit antiserum against alglucosidase alfa as a positive control. After washing of the plates, 50 μL conjugate was added to each well: polyclonal goat-anti-human-[IgG, IgA and IgM]-HRP (Acris) in a 20,000-fold dilution for human sera, and goat-anti-rabbit-IgG-HRP (Sigma) in a 10,000-fold dilution for rabbit serum. After 1 h incubation at room temperature followed by thorough washing, 100 μL Tetramethylbenzidine Microwell Peroxidase substrate (Kirkegaard and Perry Laboratories, Maryland) was added, and the plates were incubated for 10 min. The colorimetric reaction was stopped by the addition of 100 μL 1 M phosphoric acid (H_3_PO_4_). Absorbance was measured at 450 nm with a spectrophotometer (Thermo Electron Corporation, Vantaa, Finland). The maximal dilution at which absorbance was at least twice that of the negative control was taken as titer.

An antibody titer assay based on immunoprecipitation of alglucosidase alfa was used as second semi-quantitative method (de Vries et al 2010).

#### Inhibition of alglucosidase alfa uptake

Fibroblasts from a patient homozygous for the 525delT mutation fully deficient in acid α-glucosidase production were seeded in 24-well tissue-culture plates and maintained at 37°C in Ham’s F10 medium supplemented with 10 % FCS and antibiotics. To measure the uptake of alglucosidase alfa, we added Pipes to the medium in a final concentration of 3 mM to make the medium slightly acidic (pH 6.8). The enzyme was added in an amount equivalent to 200 nmol MUGlc/h per 200 μL medium. Finally, 20 μL of the patients’ sera were added. The acid α-glucosidase activity in the medium was measured directly after enzyme addition and prior to cell harvest. Uptake of alglucosidase alfa was measured in cell homogenates. MUGlc was used as substrate.

### Clinical assessments

The clinical outcome was evaluated by analyzing survival, ventilator use, gross motor function, and cardiac dimensions at baseline and at regular intervals; the latter by 2D-guided M-mode echocardiography. The LVMI was calculated as a measure for hypertrophic cardiomyopathy. Motor development was assessed using the Alberta Infant Motor Scale (AIMS) (Piper and Darrah 1994).

Safety assessments included the monitoring of infusion-associated reactions (IARs). All adverse events that were judged to be possibly, probably or definitely related to ERT were considered IARs.

### Statistical analysis

Due to limitations in patient numbers, the clinical data was summarized using descriptive statistics. All patients were followed-up for at least 4 years after the start of ERT, or until death. Data were analyzed using SPSS for Windows version 20, SPSS Inc., Chicago, IL.

## Results

### Patients

This study describes 11 patients (five boys and six girls) with classic infantile Pompe disease (Table 1). All had symptoms before the age of 3.5 months; nine started ERT before the age of 4 months. Two patients started ERT at the ages of 7 and 8 months when they were severely affected. All 11 patients had cardiac hypertrophy, less than 1 % of average normal acid α-glucosidase activity in cultured fibroblasts, and a severe mutation in both *GAA* alleles (Table 1). They were treated with ERT for 0.3 to 13.7 years (median duration 5.6 years).

**Table 1:** Baseline characteristics

Parameters at start of ERT							
Pt	Gender	Ethnic origin	Age (months)	Resp. function	AIMS score	LVMI in SD	Genotype
1	M	Turkish	0.1	OS	N	+5	c.1460 T > C
							c.1460 T > C
2	F	Caucasian	0.5	OS	N	+22	c.2481 + 102_2646 + 31del
							c.2481 + 102_2646 + 31del
3	M	Caucasian	1.2	NS	A	+15	c.1933G > T
							c.525delT
4	M	Turkish	1.9	NS	N	+30	c.2741delinsCAG
							c.2741delinsCAG
5	M	Caucasian	2.2	NS	N	+10	c.2481 + 102_2646 + 31del
							c.525delT
6	F	Caucasian	2.4	NS	A	+23	c.2481 + 102_2646 + 31del
							c.2481 + 102_2646 + 31del
7^a^	F	Caucasian	3.0	NS	A	+31	c.525delT
							c.525delT
8	F	Caucasian	3.6	OS	A	+24	c.378_379del
							c.525delT
9^a^	M	Caucasian	3.8	NS	N	+14	c.2481 + 102_2646 + 31del
							c.1799G > A
10^a^	F	Caucasian	7.2	IV	A^b^	+18	c.1115A > T
							c.525delT
11^a^	F	Caucasian	8.3	OS	A^b^	+68	c.1913G > T
							c.1548G > A
	Male 45 %	Caucasian 81 %	Median 3.0	Ventilated 9 %		Median +22	

*Pt* patient, *M* male, *F* female, *Resp* respiratory, *OS* oxygen via nasal cannula, *NS* no respiratory support, *IV* invasive ventilation, *N* within normal range of healthy peers, *A* atypical motor development (AIMS score ≤ −2SD), *LVMI* left ventricular mass index, *SD* standard deviation

^a^These patients initially received recombinant human acid α-glucosidase from transgenic rabbits

^b^Paresis arms and paralysis legs

### CRIM status

‘CRIM status’ in this study is defined as the ability to detect any acid α-glucosidase with immunological methods in either the patients’ cultured fibroblasts or in HEK293 cells in which mutated *GAA* constructs with the patients’ mutations were expressed. A CRIM-positive status indicates that molecular forms of acid α-glucosidase could be detected. A CRIM-negative status indicates that these were undetectable.

**Fig. 1:**
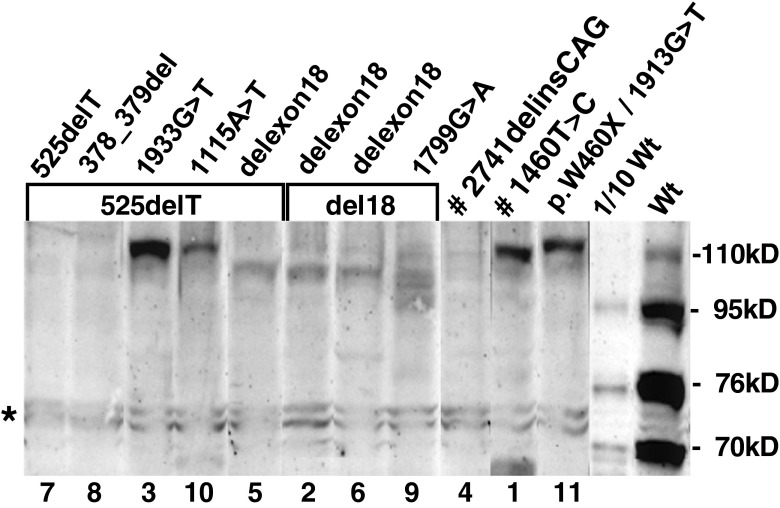
Molecular forms of acid α-glucosidase in fibroblasts. Cell homogenates were prepared as described in materials and methods. Equal amounts of protein were loaded per lane. SDS-PAGE followed by immunoblotting was used to reveal the biosynthetic forms of acid α-glucosidase. The numbers under the lanes refer to the patient numbers. The genotypes of the patients are presented above the lanes (delexon18 or del18 stands for c.2481 + 102_2646 + 31del; p.W460X stands for c.1548G > A). Wt, fibroblasts from a healthy individual; ^#^Homozygous; *Nonspecific background staining

Figure 1 presents an example of the first type of analysis in fibroblasts. Cell homogenates were analyzed by SDS-PAGE followed by immunoblotting. Compared to normal synthesis of acid α-glucosidase in fibroblasts of a healthy individual (Wt), represented by the presence of a 110 kD precursor and molecular species of 95, 76 and 70 kD, fully maturated enzyme of 76 kD was not detected in cells from any of the patients. However, the 110 kD precursor was detected in eight cases. In four of these cases (pts 1, 3, 10, and 11), the apparent molecular mass of the precursor was near normal. In the other four cases (pts 2, 5, 6, and 9), the mass was abnormally low due to either homozygosity or heterozygosity for the rather common exon 18 deletion (c.2481 + 102_2646 + 31del). Proteolytic conversion of the 110 kD precursor to the 95 kD intermediate could be demonstrated only in cells of patient 9. Three patients did not have any trace of acid α-glucosidase due to either homozygosity for c.525delT (pt 7) or c.2741delinsCAG (pt 4), or to compound heterozygosity for c.525delT and c.378_379del (pt 8). Based on the outcome of this analysis, eight of the 11 patients were designated CRIM-positive and three CRIM-negative.

**Fig. 2:**
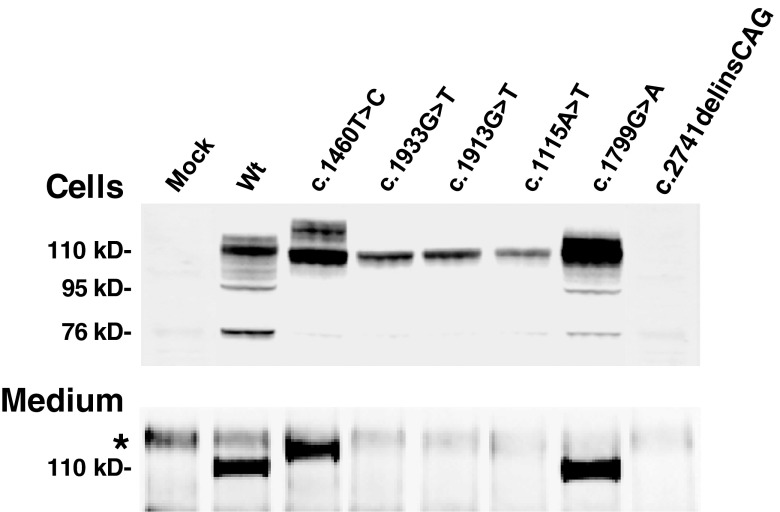
Transient expression of wildtype and mutant acid α-glucosidase cDNA constructs. Hek293T cells were transfected with wildtype (Wt) and mutant cDNA constructs to study abnormalities in the synthesis and post-translational modification of acid α-glucosidase. To visualize the molecular species, the same procedure was used as in Fig. 1. *Nonspecific background staining

As an alternative approach determining the patients’ CRIM status, we introduced the mutations in a *GAA* cDNA expression construct and studied their isolated effect on acid α-glucosidase synthesis and maturation in HEK293T cells (Fig. 2). Transient expression of the five missense mutations identified among the patients in this study revealed synthesis of the 110 kD acid α-glucosidase precursor with either normal (c.1933G > T, c.1913G > T, and c.1115A > T) or slightly higher than normal (c.1460 T > C and c.1799G > A) molecular mass. The c.1799G > A and c.1460 T > C encoded precursors were also clearly present in the culture medium, but the c.1933G > T, c.1913G > T, and c.1115A > T encoded precursors were not (Fig. 2; medium). Figures 1 and 2 both illustrate that the conversion from 110 kD to 95 kD is not blocked completely in the c.1799G > A construct; even the 76 kD form was detectable.

Transient expression of c.2741delinsCAG did not reveal any acid α-glucosidase species (Fig. 2; cells) and also this finding is consistent with the lack of acid α-glucosidase expression in fibroblasts of the patient who is homozygous for this mutation (Fig. 1). None of the mutagenized cDNA constructs led to expression of acid α-glucosidase activity in the transfected cells nor in the medium.

On the basis of these two types of analyses, patients 4, 7, and 8 were designated CRIM-negative; the other eight patients were designated CRIM-positive.

### Antibody titers

All patients developed antibodies to alglucosidase alfa (Supplemental Fig. 1a, Table 2). Over 4 years of treatment the median peak antibody titer (ELISA) was 1:31,250 (range 1:1250–1:156,250). These titers were reached within 1 year of ERT. Both low and high antibody titers were measured in CRIM-positive (median 1:18,750, range 1:1250–1:156,250) and CRIM-negative patients (median 1:31,250, range 1:6250–1:156,250). The peak antibody titers tended to be higher in patients who had started ERT relatively late (Table 2). Notably, none of the patients who received ERT before the age of 2 months developed titers above 1:6250.

**Table 2:** CRIM status, antibody titer, and clinical outcome

	Age			Antibody titer				Clinical outcome at 4 years of ERT^b^					
Pt	At start of ERT (months)	Current (years)	CRIM status	Peak titer through 4 years of ERT	Titer at 4 years of ERT^b^	Last titer (years after start of ERT)		Alive	Invasive ventilation^c^		Motor status	LVMI in SD	Dose of ERT
1	0.1	5.6	+	1:6,250	1:1,250	1:6,250	(5.0)	Y	Y	2.7 (1:250)	Sitting^e^	−0.6	40^f^
2	0.5	8.0	+	1:1,250	1:250	1:250	(7.1)	Y	N		Walking	−0.7	20
3	1.2	8.5	+	1:6,250	1:1,250	1:6,250	(8.0)	Y	N		Walking	−1.5	40^f^
4	1.9	*4.4* ^a^	−	1:6,250	1:50	1:50	(3.4)	Y	Y	2.0 (1:6,250)	Sitting^e^	+0.9	20
5	2.2	5.2	+	1:31,250	1:31,250	1:31,250	(4.0)	Y	N		Sitting	+1.1	40^f^
6	2.4	4.1	+	1:31,250	1:31,250	1:31,250	(3.0)	Y	N		Walking	+3.7	40
7	3.0	*4.3* ^a^	−	1:156,250	1:156,250	1:156,250	(3.8)	Y	Y	2.2 (1:31,250)	Sitting	+4.4	40^f^
8	3.6	*0.6* ^a^	−	1:31,250	1:31,250	1:31,250	(0.3)	N	N		Minimal motor gain	+28.6	20
9	3.8	14.1	+	1:6,250	1:250	1:6,250	(12.8)	Y	N		Walking	+0.4	40^f^
10	7.2	14.3	+	1:31,250	1:6,250	1:156,250	(12.9)	Y	Y	0.6^d^	Tetraplegic	+4.9	40^f^
11	8.3	14.3	+	1:156,250	1:156,250	1:156,250	(11.5)	Y	Y	0.9 (1:250)	Tetraplegic	+4.5	40^f^

*Pt* Patient number, *ERT* enzyme-replacement therapy, *CRIM* cross reactive immunological material, *LVMI* left ventricular mass index, *SD* standard deviation

^a^Age at death

^b^Last available data are presented if patient did not yet receive 4 years of ERT

^c^Age in years (antibody titer around that time)

^d^Patient was ventilator dependent before start of ERT

^e^Patients lost ability to walk

^f^Dose was augmented to 40 mg/kg/week during treatment

Six patients (pts 5–8, 10 and 11) developed titers ≥ 1:31,250 (Table 2); titers remained high in five cases and declined temporarily to 1:6250 in one case (pt 10). The antibody titers of the other five patients remained ≤ 1:6250 over the entire study period; one of these patients was CRIM-negative and his titer eventually declined to 1:50. There was no apparent relationship between the titer and the dose of ERT, nor between the titer and the use of recombinant human acid α-glucosidase from either rabbit milk or CHO cells (results not shown).

An immunoprecipitation assay was used as an alternative method for determining the antibody titer, for which we selected three patients whose respective ELISA titers were 1:1250, 1:6250, and 1:156,250. The results are shown in Supplemental Fig. 2 and are consistent with the outcome of the ELISA assay in that both methods discriminate similarly between a low, intermediate and high titer.

#### Inhibition of alglucosidase alfa uptake

To measure the effect of antibodies on the uptake of alglucosidase alfa, we used sera of all patients at the time that their antibody titer was highest (Supplemental Table 1). Alglucosidase alfa and patients’ sera were added together to the culture media of fibroblasts from a CRIM-negative infant. No effects on enzyme activity or uptake of alglucosidase alfa were observed when antibody titers were ≤ 1:6250. Alglucosidase alfa activity in the medium was substantially inhibited (26–50 % loss of enzymatic activity) by one of three sera with a titer of 1:31,250 and by all three sera with a titer of 1:156,250. Only the three sera with the highest titer (1:156,250) resulted in inhibition (24–42 %) of alglucosidase alfa uptake.

### Clinical outcome over 4 years of ERT

#### Survival and ventilator-free survival

One of the 11 patients was ventilator dependent before the start of ERT. Over 4 years of ERT, four more patients required invasive ventilation (at ages 0.9, 2.0, 2.2, and 2.7 years; Table 2) and three patients died (at ages 0.6, 4.3, and 4.4 years). The last antibody titers measured before the patients’ deaths or respiratory insufficiency ranged from 1:50 to 1:31,250. The median peak antibody titer of these patients was 1:31,250 (range 1:6250–1:156,250); the median peak antibody titer of the five patients who are still alive and ventilator-free at the time of reporting was 1:6250 (range 1:1250–1:31,250). All three CRIM-negative patients died. All eight CRIM-positive patients are alive; two of them became ventilator dependent during treatment.

#### Motor outcome

Supplemental Fig. 1C depicts motor development over time as measured by AIMS. Over 4 years of ERT, eight patients achieved important motor milestones. Six learned to walk and two learned to sit, but three made minimal motor gains. The median peak titer of the six patients who learned to walk was 1:6250 (range 1:1250–1:31,250) against 1:31,250 (range 31,250–156,250) for the patients who did not learn to walk. One of the three CRIM-negative patients and five of the eight CRIM-positive patients learned to walk.

Patients 1 and 4 lost their ability to walk after developing respiratory insufficiency. Their antibody titers never rose above 1:6250. One of them was CRIM-positive, the other CRIM-negative. One other CRIM-positive patient with a peak titer of 1:31,250 temporarily lost the ability to attain a sitting position after a respiratory syncytial virus (RSV) infection at the age of 1.3 years (Supplemental Fig. 1c, pt 5). None of the patients with a longer than 4 years follow-up lost or gained motor milestones beyond 4 years of ERT.

#### Cardiac dimensions

At the start of ERT, all patients had hypertrophic cardiomyopathy. Over 4 years of ERT, the LVMI decreased significantly in all but one patient who died 4 months after ERT was initiated (Supplemental Fig. 1b). Six of the 11 patients had a normal LVMI at 4 years of ERT.

The median peak antibody titer of the patients with a normal LVMI after 4 years of ERT was 1:6250 (range 1:1,250–31,250) against 1:31,250 (range 1:6250–1:156,250) for those whose LVMI was increased. The LVMI remained above the reference value in all three CRIM-negative patients, against two of the eight CRIM-positive patients. Beyond 4 years of ERT, no major changes in LVMI were observed.

#### Infusion-associated reactions

Over 4 years of ERT, ten of the 11 patients (82 %) experienced one or multiple IARs. The first IARs started 0.4–24.5 months after the start of ERT and comprised the following signs and symptoms: General malaise, hypoxia, bronchospasm, dyspnea, tachycardia, bradycardia, hypotension, cyanosis, sweating, fever, vomiting, flushing, and exanthema. All IARs could be controlled by slowing the infusion rate, with or without the administration of premedication.

The only patient who did not develop IARs was CRIM-positive and had the lowest peak antibody titer over the whole period of reporting.

## Discussion

Several studies have shown that antibody formation can occur in patients receiving recombinant human proteins for therapy (Wang et al 2008; Kishnani et al 2010; Banugaria et al 2011; Patel et al 2012). Our patients with classic infantile Pompe disease who were treated with alglucosidase alfa showed various immune response patterns. Low and high titers were measured in CRIM-negative and CRIM-positive patients alike. While thorough statistical analysis was prevented by the small sample size and the heterogeneity of our patient population, we noticed that patients who did not learn to walk had a relatively high titer. We also noticed that patients who had started ERT before the age of two months tended to have lower titers than those who had started later. Notably, all CRIM-positive patients were still alive at the time of reporting, whereas all CRIM-negative patients had died.

### *GAA* genotypes and CRIM status

The quality and quantity of acid α-glucosidase in Pompe disease depends on the *GAA* genotype. Ten different mutations were identified, all causing complete loss of acid α-glucosidase activity.

The commonly used procedure to determine the CRIM status is immunoblotting and normally reveals four distinct acid α-glucosidase species that separately and collectively are called CRIM. In healthy people (Fig. 1, Wt), the long-lived 76 and 70 kD species comprise approximately 80 % of the total CRIM. In patients, however, the synthesis of acid α-glucosidase can be totally lost, which results in a CRIM-negative status. More often, the synthesis is derailed, and CRIM consists mainly of the 110 kD precursor (e.g., Fig. 1, pts. 1 and 3) or a modified derivative thereof (Fig. 1, pts. 2 and 6). As it stands, the CRIM-negative status is well defined, but the CRIM-positive status encompasses a collection of qualitatively and quantitatively abnormal immunoblot profiles.

By and large, a patient’s CRIM status can be predicted on the basis of its genotype (Bali et al 2012). Most non-sense mutations and frame-shift mutations lead to mRNA decay and a CRIM-negative status (e.g., 525delT in pt. 3), truncated protein species are sometimes detectable by immunoblotting (e.g., c.2481 + 102_2646 + 31del in pts. 1, 7, 10, and 11), and thus lead to a CRIM-positive status. Although the effect of missense mutations is hard to predict, they usually lead to a CRIM-positive status.

Information on the effect of missense mutations can be obtained by transient expression (Fig. 2), which is especially informative for the stage at which mutant forms of acid α-glucosidase are degraded. For instance, the c.1460 T > C or c.1799G > A encoded acid a-glucosidase precursors must have traversed the Golgi complex since they are secreted into the medium, whereas the c.1833G > T, c1913G > T and c1115A > T encoded precursors do not appear in the medium (Fig. 2) and are apparently degraded while passing through the ER/Golgi complex. By the current definition of CRIM, these five missense mutations lead to a CRIM-positive status, but obviously do not represent one and the same CRIM-positive condition.

Our application of both methods to determine the CRIM status resulted in the same outcome: eight patients are CRIM-positive and three are CRIM-negative. Notably, the patient homozygous for c.2741delinsCAG was previously designated CRIM-positive (Hermans et al 1998; Banugaria et al 2011).

### CRIM status and antibody titer

Antibody formation is a natural response to foreign invading proteins. Thus, ERT is prone to evoke an immune response in CRIM-negative patients, but not necessarily in all CRIM-positive patients. Patients with Pompe disease receiving ERT respond roughly according to these principles. Analysis of the immune response in 34 treated infants showed a strong tendency toward higher and sustained antibody titers in CRIM-negative compared to CRIM-positive infants (Kishnani et al 2010). However, CRIM-positive patients can also develop high titers (Banugaria et al 2011), and CRIM-negative patients low titers (Abbott et al 2011; Al Khallaf et al 2013). Our study in 11 infants has led to similar findings. The highest peak antibody titers were measured in four of the eight CRIM-positive patients and in two of the three CRIM-negative patients. The antibody titer of the third CRIM-negative patient did not exceed 1:6250 and spontaneously regressed to 1:50 after 3 years of ERT. There were two CRIM-positive patients with the same *GAA* genotype; one developed a relatively high titer, and the other a relatively low titer.

Although the number of patients in our study is small, we can firmly conclude that the CRIM status alone predicts neither the level nor the duration of the immune response. This may relate to the imprecise definition of CRIM status, which does not describe the amount, the conformation or the location of the endogenous acid α-glucosidase. For instance, secretion of the 110 kD precursor as opposed to intracellular degradation might reduce the immune response and contribute to the relatively low antibody titers in patients 1 and 9. Notably, adult patients with a considerable amount of normally structured and catalytically active acid α-glucosidase can also develop high titers (van der Ploeg et al 2010; de Vries et al 2010; Patel et al 2012; Regnery et al 2012). Altogether, we conclude that genotype and CRIM status are of limited value in predicting the height of the immune response.

As previously suggested (van Gelder et al 2012; Al Khallaf et al 2013), our study indicates that the age at start of ERT might play a role in the immune response since none of the patients who started ERT before 2 months of age developed titers > 1:6250. There are at least two plausible explanations for this: First, the neonatal immune system is immature, and very early administration of ERT might induce tolerance (Brooks 1999; Dierenfeld et al 2010). Second, the ‘danger model’ suggests that the immune system needs alarm signals from injured tissues to be activated. At a less advanced stage of disease these signals will be weaker (Matzinger 2002). Other models may equally apply.

### Consequences of antibody formation

Depending on their binding sites antibodies can inhibit catalytic function, block binding to the mannose 6-phosphate receptor and prevent uptake, or otherwise misdirect the protein to macrophages and neutrophils (Brooks et al 2003; Wang et al 2008). Antibodies and antigen can also form immune complexes and trigger a cascade of adverse events (Hunley et al 2004).

In a previous case-report about an adult patient with Pompe disease we have calculated how the concentration of alglucosidase alfa specific antibodies relates to the concentration of alglucosidase alfa during enzyme infusion (de Vries et al 2010). In the present study we have applied the same arithmetic method to infants. For example, patient 11 had an ELISA titer of 1:156,250 at a certain point of treatment. At that time the corresponding titer as measured by immune-precipitation was 40 nmol MUGlc/h.μL (Supplemental Fig. 2) implying that 1 mL of the patient’s serum contained enough antibodies to bind 0.13 mg alglucosidase alfa (de Vries et al 2010). As a child receives 0.24 mg of alglucosidase alfa per mL blood (based on 20 mg/kg and a circulating blood volume of 80 mL/kg), antibodies may bind as much as 54 % of the administered enzyme. By contrast, if the titer is only 4 nmol MUGlc/h.μL, corresponding with an ELISA titer of 1:6250, the circulating antibodies can bind only 5 % of the administered aglucosidase alfa.

Although these estimates are crude they indicate that ELISA titers of 1:6250 and lower probably have no clinical significance, whereas titers of 1:31,250 and higher may counteract ERT at a dose of 20 mg/kg. Accordingly, titers above 1:60,000 are expected to counteract ERT at a dose of 40 mg/kg. Our arithmetic estimates of what should be considered a high titer and what should be considered a low titer remarkably correspond to the cut-off value of 1:51,200 chosen by Banaguria et al (Banugaria et al 2011).

Antibodies can impede the effect of ERT in several ways. In four cases we observed a decrease of catalytic activity suggestive for binding of antibodies to the enzyme’s active site. Uptake was inhibited in three cases. In one case this was due to a combination of inactivation and uptake inhibition possibly by steric hindrance of the ligand-receptor binding. Of note, the effect of antibodies not only depends on the binding sites but also on the stoichiometry of antibodies and alglucosidase alfa in the experimental setting; changing the concentration of one of the two components can tip the balance. Whereas neutralizing antibodies were reported to be more common in CRIM-negative than in CRIM-positive patients (Kishnani et al 2010; Banugaria et al 2011), in our study their presence was not related specifically to the patients’ CRIM status but to the patients’ antibody titer.

It has been shown that high antibody titers in CRIM-negative as well as CRIM-positive patients are associated with shorter ventilator-free survival (Kishnani et al 2010; Banugaria et al 2011). In several adults with Pompe disease, a high titer was also accompanied by poor response to ERT (de Vries et al 2010; Patel et al 2012). Although our study group was too small and too varied to permit statistical analysis, patients with higher titers tended to attain fewer motor milestones. However, with respect to ventilator-free survival, only two of the six patients who developed respiratory insufficiency or died had a titer of ≥ 1:31,250 at the time that these events occurred. Two other patients had low titers, but nevertheless developed respiratory insufficiency at the age of 2. Two patients became ventilator dependent just before or soon after the start of ERT which we ascribe to their advanced disease at the start of ERT.

### Conclusions and challenges

The outcome of classic infantile Pompe patients is determined by many factors, among which are the age and disease severity at initiation of ERT, the patient’s genotype, and the height of the antibody response. It is beyond question that high and sustained antibody titers need to be prevented in order to achieve a good response to ERT (Kishnani et al 2010; de Vries et al 2010; Banugaria et al 2011). Our study indicates that the negative effect of antibodies starts at an antibody titer of approximately 1:31,250 when the recommended dose of 20 mg/kg is used. Very early start of ERT (<2 months), achievable by neonatal screening, may help to keep the antibody titers low. Recent publications indicate that immune tolerance can be successfully induced by a combination of rituximab, methotrexate, and intravenous immunoglobulins (Mendelsohn et al 2009; Messinger et al 2012; Banugaria et al 2013b), a regimen likely to be most effective when used prophylactically (Lacaná et al 2012; Banugaria et al 2013a). As the patients’ genotype and related CRIM status alone are obviously not the sole determinants for the patients’ immune response, identification of other triggering factors remains a challenge. Though the number of patients is very small, it occurred to us that the three CRIM-negative patients in our study had an early demise while all CRIM-positive patients survived. If the difference is not made solely by the CRIM-status or antibody titer, might it be that CRIM-positive patients can have a tiny bit of acid α-glucosidase activity in their tissues that we cannot detect with our current methods?

## Electronic supplementary material

Below is the link to the electronic supplementary material.

Supplemental Fig. 1 Antibody titers, LVMI, and AIMS score over time. Antibody titers to alglucosidase alfa as measured by ELISA in CRIM-positive and CRIM-negative patients (a). LVMI (b) and raw AIMS scores (c) (GIF 24 kb)

High resolution image (EPS 98 kb)

Supplemental Fig. 2 Antibody titer by immunoprecipitation. A fixed amount of alglucosidase alfa was incubated with different volumes of patients’ sera: Patient 2 (■); patient 10 (▲); patient 11 (●); serum of a healthy individual (−−); and rabbit serum raised against alglucosidase alfa (∙∙∙). Antibody-bound enzyme was precipitated with protein A sepharose beads, and the activity remaining in the supernatant was measured with MUGlc. *Corresponding ELISA titer (GIF 14 kb)

High resolution image (EPS 82 kb)

Supplemental Table 1 Uptake of alglucosidase alfa by cultured fibroblasts (DOCX 15 kb)

## Abbreviations

ERT: enzyme-replacement therapy
CRIM: cross reactive immunological material
LSDs: lysosomal storage disorders
MPS: mucopolysaccharidoses
*GAA*: acid α-glucosidase gene
LVMI: left ventricular mass index
FCS: fetal calf serum
PBS: phosphate-buffered saline
MUGlc: 4-methylumbelliferyl-α-D-glucopyranoside
ELISA: enzyme-linked immunosorbent assay
BSA: bovine serum albumin
AIMS: Alberta Infant Motor Scale
IAR: infusion-associated reaction.
