# Supplementary figures and images for: Enzyme therapy and immune response in relation to CRIM status: the Dutch experience in classic infantile Pompe disease
Source: J Inherit Metab Dis. 2014 Apr 9;38(2):305–14. doi: 10.1007/s10545-014-9707-6 (PMC4341007; doi:10.1007/s10545-014-9707-6)

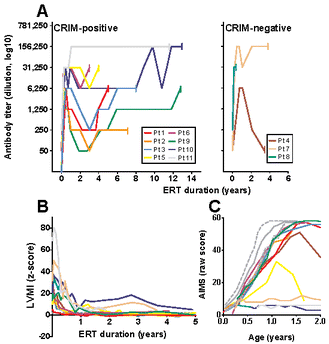

Supplement: Supplementary file 1 — Antibody titers, LVMI, and AIMS score over time. Antibody titers to alglucosidase alfa as measured by ELISA in CRIM-positive and CRIM-negative patients (a). LVMI (b) and raw AIMS scores (c) (GIF 24 kb) [file 10545_2014_9707_Fig3_ESM.gif]

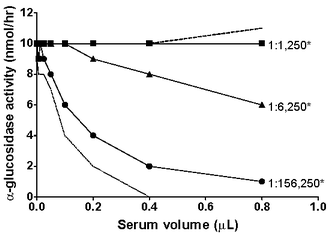

Supplement: Supplementary file 3 — Antibody titer by immunoprecipitation. A fixed amount of alglucosidase alfa was incubated with different volumes of patients’ sera: Patient 2 (■); patient 10 (▲); patient 11 (●); serum of a healthy individual (−−); and rabbit serum raised against alglucosidase alfa (∙∙∙). Antibody-bound enzyme was precipitated with protein A sepharose beads, and the activity remaining in the supernatant was measured with MUGlc. *Corresponding ELISA titer (GIF 14 kb) [file 10545_2014_9707_Fig4_ESM.gif]
