# Supplementary material for: Enzyme therapy and immune response in relation to CRIM status: the Dutch experience in classic infantile Pompe disease
Source: J Inherit Metab Dis. 2014 Apr 9;38(2):305–14. doi: 10.1007/s10545-014-9707-6 (PMC4341007; doi:10.1007/s10545-014-9707-6)
Supplement: Supplementary file 5 — Uptake of alglucosidase alfa by cultured fibroblasts (DOCX 15 kb) [file 10545_2014_9707_MOESM3_ESM.docx]

**Supplemental Table 1**

**Uptake of alglucosidase alfa by cultured fibroblasts**

|  | **Activity compared**  **to control (%)** | | **Antibody titer** |
| --- | --- | --- | --- |
|  | **Medium** | **Cell^a^** |  |
| **Pt 1** | 93 | 90 | 1:6,250 |
| **Pt 2** | 97 | 111 | 1:1,250 |
| **Pt 3** | 92 | 85 | 1:6,250 |
| **Pt 4** | 95 | 90 | 1:6,250 |
| **Pt 5** | 88 | 112 | 1:31,250 |
| **Pt 6** | **74** | 83 | 1:31,250 |
| **Pt 7** | **58** | **24** | 1:156,250 |
| **Pt 8** | 81 | 91 | 1:31,250 |
| **Pt 9** | 93 | 96 | 1:6,250 |
| **Pt 10** | **50** | **42** | 1:156,250 |
| **Pt 11** | **60** | **41** | 1:156,250 |
| **Rabbit antiserum** | **49** | **1** | >1:156,250 |
| **Control** | 100 | 100 |  |
| **Blanco** |  | 1 |  |

Pt, Patient; Control, Serum from healthy individual

^a^ Mean of two experiments

Figures in bold refer to substantial inhibition of alglucosidase alfa activity or uptake.
